# Chronic kidney disease in Polish elderly population aged 75+: results of the WOBASZ Senior Survey

**DOI:** 10.1007/s11255-016-1477-7

**Published:** 2016-12-17

**Authors:** Łukasz Zdrojewski, Ewa Król, Bolesław Rutkowski, Walerian Piotrowski, Andrzej Pająk, Wojciech Drygas, Tomasz Zdrojewski

**Affiliations:** 1 Department of Nephrology, Transplantology and Internal Medicine, Medical University of Gdansk, ul Dębinki 7, 80-952 Gdańsk, Poland; 2 Department of Epidemiology, Cardiovascular Disease Prevention and Health, The Cardinal Stefan Wyszynski Institute of Cardiology, Warsaw, Poland; 3 Department of Clinical Epidemiology and Population Studies, Institute of Public Health, Jagiellonian University Medical College, Kraków, Poland; 4 Department of Preventive Medicine and Medical Education, Medical University of Gdansk, Gdańsk, Poland

**Keywords:** Chronic kidney disease, Population survey, Elderly, eGFR, Diabetes, Hypertension, Cardiovascular disease

## Abstract

**Purpose:**

Kidney filtration decreases with age, which results in an increased frequency of chronic kidney disease (CKD) in the elderly population. The purpose of the study was to assess the prevalence and epidemiology of CKD in the Polish elderly population.

**Methods:**

A representative sample of the Polish elderly population, composed of 918 people (F 452, M 466) in the age of ≥75 years, was chosen. All participants had their history, anthropometric measures and biochemical parameters (creatinine, fasting glucose, complete cholesterol) evaluated. CKD was diagnosed when eGFR was <60 ml/min/1.73 m^2^. The comorbidities, anthropometric and social factors connected with the onset of CKD were also analyzed.

**Results:**

The prevalence of CKD in the analyzed population was 26.9% (F 32.0%, M 15.8%), which gives an estimated number of 495,590 (95% CI 396,363–594,817) patients in the study subpopulation. The majority of these people were in the G3A category—70.1%, while the remaining fell under the G3B—25.7%, G4—3.1% and G5—1.1% categories. Disease awareness among the participants was found to be at 17%. Arterial hypertension (AH) was more frequent in people with CKD (91.0 vs. 80.3%, *P* < 0.001), whereas diabetes mellitus (DM) prevalence was comparable in both CKD and non-CKD groups (11.7 vs. 11.4%, ns). In the examined group, DM had no influence on the frequency of CKD. In contrast, the presence of cardiovascular diseases substantially increased the chances of developing CKD (OR 1.87, *P* < 0.05).

**Conclusion:**

1. The prevalence of CKD in the Polish elderly population was 26.9%. 2. Awareness of CKD is low. 3. DM, increasing age and AH did not increase the risk of CKD. 4. Coexistence of cardiovascular diseases increased the risk of having CKD.

## Introduction

Chronic kidney disease (CKD) is a disease characterized by a decrease in renal filtration function and/or damage to the kidney structure. This disease was precisely defined in guidelines published in 2012 by the international group, “Kidney Disease—Improving Global Outcome (KDIGO) [1]”. This revised definition includes the previously known criteria which divided CKD into five stages of disease progression, based on the estimated value of glomerular filtration rate (eGFR), additionally appended with the level of albuminuria intensity. The severity of CKD assessed in this manner is therefore linked to the risk of progression to end-stage renal disease, as well as occurrence of cardiovascular complications [2, 3].

Based on previous population studies, it has been shown that the frequency of CKD prevalence increases with age [4, 5]. It has also been proven that in the population of elderly people, the prevalence is high and reaches 19–29% in European and American populations [6–10]. However, there is still a fierce discussion on whether the high prevalence of CKD in the elderly population is indeed a pathological state or a result of the general physiological aging of the organism. What is more, the traditional criteria of CKD diagnosis and classification based on eGFR values are calculated on the basis of formulae not validated for the discussed population; therefore, it does not take into account neither the physiological changes connected with aging nor pathologies of elderly, such as sarcopenia or frailty syndrome. Taking the above into consideration, there is an ongoing discussion on whether it is legitimate to apply the same diagnostic criteria for elderly people as that of the remaining younger part of the population [11]. Analysis of epidemiological studies may be helpful in finding an answer to this question. Therefore, the aim of this paper is to assess the prevalence of CKD in the Polish population of elderly people of 75 years and above, who took part in the WOBASZ Senior Survey.

## Methods

The WOBASZ Senior Survey is a cross-sectional survey conducted on a representative sample of Polish people in the age of ≥75 years. The primary objective of the survey was the assessment of cardiovascular diseases in this population [12]. A two-staged sampling scheme was used, stratified according to provincial and district categories (2 small districts, 2 medium and 2 large districts from every province). In the first stage, streets (in municipalities) or villages (in communes) were drawn. In the second stage of sampling, people who met the sex and age criteria were drawn from those registered in given streets or villages. The sample size was selected in proportion to the percentage of the Polish population aged ≥75. The draw was conducted in the Department of National Registers at the Ministry of Internal Affairs and Administration (MSWiA). A total of 1070 men and 1084 women were drawn. After verification (having subtracted those unavailable for the study), the final sample was 755 men and 704 women. 508 men and 502 women gave their written consent for the participation in the study. The final response rate was 67.3% among men and 71.3% among women. Comprehensive results which included a questionnaire, laboratory results and anthropometric measurements were obtained from 918 people (F 452, M 466). The study, which consisted of taking medical history, answering a sociodemographic questionnaire, anthropometric measurements and blood sample collection was conducted by trained research teams in accordance with the protocol prepared by the regulatory team. Laboratory tests were performed in a central laboratory with international standardization. Serum creatinine concentration was measured using reagents from the Roche Company through the Jaffe buffer kinetic method on the Integra 800 analyzer.

Renal function was determined using eGFR and calculated using the simplified MDRD equation (MDRD4) [13–15]. CKD was diagnosed when eGFR was lower than 60 mL/min/1.73 m^2^. A simplified division of CKD into stages was applied, leaving out G1 and G2 stages. Stages G3a (eGFR 45–59 mL/min/1.73 m^2^), G3b (30–44), G4 (15–29), G5 (<15) were diagnosed, in correspondence with the former definition of chronic kidney failure [1, 16].

Diabetes mellitus (DM) was noted when a patient declared that it had been previously diagnosed or if fasting glucose was ≥126 mg/dL. The respondent was diagnosed with arterial hypertension (AH) when one declared regular intake of hypotensive drugs or when the average value of two arterial blood pressure measurements taken at two separate visits was ≥140/90 mmHg. Additional information concerning the coexistence of other diseases, awareness of kidney disease, proteinuria, nocturia, socioeconomic status and the respondent’s educational status was obtained through the use of a questionnaire. Statistical weights were applied to improve adjustment of the age and sex structure of the study population to match that of the general population aged ≥75 years, on the basis of data published by the Central Statistical Office. Statistical analysis was performed using IBM SPSS statistics version 19, Complex Samples Module software. Statistical significance was established by Chi-squared test, and logistic regression analysis was used to establish confidence intervals (CIs) and odds ratios (ORs).

## Results

### Chronic kidney disease

The average age of the examined population was: men (M) 78.9 years, women (F) 79.2. Successively, the average age of the group diagnosed with CKD was 80.5 (M) and 80.1 (F). Using the above-mentioned diagnostic criteria, chronic kidney disease with eGFR < 60 mL/min/1.73 m^2^ (≥G3a) was diagnosed in 196 people (129 F, 67 M). In the 75–79 years subgroup, there were 111 people, 80–84 years—60 people, 85–89 years—21 people, and in the 90+ group they were 4 people. After extrapolating these results to the Polish population with a use of the above-mentioned methods, the prevalence of CKD in those aged 75+ was 26.9%, with a higher prevalence in women (32.0%) than in men (15.8%), *P* < 0.001 (Table 1). A subsequent increase in the incidence of CKD was observed with increase in age: in the interval 75–79 years—24.2% (F 29.6%; M 14.4%), 80–84 years—29.4% (F 34.1%; M 15.2%), 85–89 years—30.4% (F 35.3%; M 21.2%), ≥90 years—37.2% (F unknown; M 31.4%).

**Table 1:** CKD prevalence (eGFR < 60 ml/min/1.73 m^2^) in polish elderly population ≥75 standardized to resident population depending on applied diagnostic criteria

Age (years)	Prevalence of eGFR < 60 mL/min/1.73 m^2^									
	>75 (the studied population)		75–79		80–84		85–89		>90	
	%	95% CI	%	95% CI	%	95% CI	%	95% CI	%	95% CI
F + M	26.9	23.1–30.9	24.2	20.2–28.7	29.4	23.2–36.5	30.4	21.5–41.2	37.2	9.7–76.5
F	32.0	27.7–36.7	29.6	24.6–35.1	34.1	26.5–42.6	35.3	23.0–49.8		
M	15.8	10.4–23.4	14.4	8.2–24.0	15.2	8.4–26.2	21.2	11.5–35.8	31.4	5.7–77.5

WOBASZ Senior: general population of the study: 918 (F 452, M 466), CKD population: 196 (F 129, M 67), age ≥75, MDRD4 eGFR (mL/min/1.73 m^2^)

*F* female, *M* male

We also evaluated the incidence of the various CKD stages in the population of elderly people. The majority of people diagnosed with CKD were in the G3A category—70.1%, while the remaining population fell under the G3B—25.7%, G4—3.1% and G5—1.1% categories (Table 2). In summary, based on the results above, it can be assumed that in the Polish population aged 75+, approximately 495,590 (95% CI 396,363–594,817) people met the criteria for diagnosis of chronic kidney disease.

**Table 2:** Percentage distribution of the diseased into CKD stages

CKD stage	eGFR range value	Age group (%), (95% CI)		
		≥75	75–84	≥85
G3A	60–45	70.1 (59.0–79.2)	64.5 (53.1–74.4)	5.6 (2.8–11.0)
G3B	44–30	25.7 (17.0–36.8)	19.4 (11.6–30.5)	6.3 (3.0–12.6)
G4	29–15	3.1 (1.4–6.6)	2.4 (1.0–5.8)	0.7 (0.2–2.8)
G5	≤14	1.1 (0.2–5.6)	1.1 (0.2–5.6)	–

WOBASZ Senior: general population of the study: 918 (F 452, M 466), CKD population: 196 (F 129, M 67), age ≥75, MDRD4 eGFR (mL/min/1.73 m^2^)

The awareness of kidney disease was relatively low in the study population. Only 17.0% (95% CI 10.4–26.5) of the population aged 75+ was aware of this ailment. This percentage was slightly higher in women (17.6%) than in men (15.4%), but the difference was not statistically significant.

### Coexisting diseases

Among diseases that most often coexist with CKD and simultaneously are the most often primal cause of progressive kidney function loss in the general population are arterial hypertension and diabetes. Epidemiology of the coexistence of these pathological states with CKD in the Polish population aged 18–79 years has been previously described [5]. Among the studied group in the age of ≥75 years, high prevalence of arterial hypertension (AH) was remarkable, both in people with normal and impaired kidney function (Table 3). In people with eGFR > 60 mL/min/1.73 m^2^, 80.3% had AH, whereas in those with eGFR < 60 mL/min/1.73 m^2^ AH was found in 91.0% (*P* < 0.001). In both groups, AH was more frequent in women, but these differences were not statistically significant (F 84.2% and M 73.6% vs. F 93.1% and M 82.0%). In contrast, a low prevalence of DM was observed in both people with normal and impaired kidney function. In people with eGFR > 60 mL/min/1.73 m^2^, DM was diagnosed in 11.4%, and in those with an eGFR < 60 mL/min/1.73 m^2^, DM was found in a similar percentage of people—11.7% (a statistically insignificant difference). Among diabetic patients, those with normal kidney function were predominantly males (F 10.8 vs. M 13.7%), whereas in the group with lower eGFR there was a higher percentage of women (F 13.3 vs. M 9.0%); however, statistical significance was again not demonstrated (Table 3).

**Table 3:** Characteristics of CKD and non-CKD populations

Parameter	eGFR > 60 mL/min/1.73 m^2^			eGFR < 60 mL/min/1.73 m^2^		
	F + M	F	M	F + M	F	M
Hypertension prevalence (%), (95% CI)	80.3 (76.5–83.7)	84.2 (78.5–88.6)	73.6 (68.4–78.3)	91.0 (86.4–94.1)	93.1 (87.4–96.3)	82.0 (72.2–88.8)
Diabetes mellitus prevalence (%), (95% CI)	11.4 (7.7–16.6)	10.8 (6.6–17.2)	13.7 (8.3–21.8)	11.7 (7.4–18.0)	13.3 (7.1–23.5)	9.0 (6.5–12.3)
BMI, mean value (kg/m^2^), (95% CI)	27.6 (27.0–28.2)	27.9 (27.1–28.8)	27.1 (26.4–27.8)	29.6 (28.8–30.4)	30.0 (29.1–30.9)	27.7 (26.4–27.8)
BMI (%), (95% CI)						
18.50–24.99	31,7 (27.5–36.3)	29,5 (23.7–36.0)	35,6 (30.3–41.3)	19,8 (14.1–27.2)	17,5 (10.9–26.8)	30,0 (22.4–38.9)
25.00–29.99	41.3 (36.2–46.7)	42.1 (34.7–49.8)	40.0 (34.3–46.0)	33.8 (26.2–42.2)	31.7 (22.9–42.1)	33.8 (26.2–42.2)
≥30	27.0 (22.1–32.5)	28.4 (21.4–36.7)	24.4 (19.3–30.4)	46.4 (39.4–53.6)	50.8 (42.2–59.3)	27.4 (19.5–37.0)
WtHR, mean value (95% CI)	0.90 (0.88–0.91)	0.87 (0.85–0.87)	0.96 (0.95–0.97)	0.90 (0.87–0.92)	0.89 (0.86–0.92)	0.93 (0.87–0.98)
WtHR increased (%), (95% CI)	67.1 (61.7–72.1)	61.2 (53.5–68.4)	67.1 (61.7–72.1)	67.4 (49.7–81.2)	65.2 (43.9–81.8)	76.5 (68.7–83.0)
Total cholesterol, mean value (mg/dl), (95% CI)	200 (196–204)	206 (200–212)	190 (186–194)	207 (201–213)	208 (202–215)	202 (192–211)
Total cholesterol, increased (%), (95% CI)	58.8 (53.6–63.8)	65.6 (57.8–72.5)	47.1 (41.4–52.9)	61.3 (54.2–68.0)	61.4 (53.0–69.2)	60.7 (54.2–68.0)
LDL, mean value (mg/dl), (95% CI)	129 (125–133)	132 (126–138)	124 (121–128)	133 (128–138)	133 (127–140)	132 (121–142)
LDL increased, (%) (95% CI)	65.4 (60.3–70.2)	68.0 (60.3–74.8)	61.0 (60.3–70.2)	64.4 (56.4–71.6)	63.9 (54.4–72.4)	66.4 (55.2–76.1)

WOBASZ Senior: general population of the study: 918 (F 452, M 466), CKD population: 196 (F 129, M 67), age ≥75, MDRD4 eGFR (ml/min/1.73 m^2^)

*F* female, *M* male, *BMI* body mass index (kg/m^2^), *WtHR* waist-to-hip ratio, increased WtHR: *M* > 0.9, *K* > 0.85, increased total cholesterol >190 mg/dl; increased LDL > 115 mg/dl

### Risk factors

The results of multivariate logistic regression analysis in the population aged ≥75 years differ from those obtained from younger populations for selected classical risk factors of CKD (Table 4). Clearly, increasing age is one of the major risk factors of kidney failure, yet in the population in question, it was not as strong a factor as in younger populations. It was not proven that there exists any statistical significance of increased odds ratios for decreased kidney function and the coexistence of arterial hypertension, as well as increasing values of systolic, diastolic nor mean arterial pressure. The indisputable relationship between AH and CKD observed in younger cohorts [5] was only suggested in the studied population (odds ratio OR 1.5, *P* > 0.05). It was also surprising to observe the lack of impact of coexisting DM on CKD prevalence. It was, however, noticed that the risk is increased by the presence of cardiovascular diseases (coronary heart disease, heart failure, peripheral arteries disease, stroke—OR 1.87, *P* < 0.05). The results also seemed to suggest a positive role of an increased waist-to-hip ratio (WtHR) value, although no such link was found for body mass index (BMI). Furthermore, among people with reduced eGFR, normal body mass (BMI 18.5–24.9 kg/m^2^) was less frequently observed, while obesity (BMI > 30 kg/m^2^) was more frequent in comparison with people with normal eGFR (Table 3).

**Table 4:** Multivariate logistic regression analysis estimating correlates of CKD

Variable	eGFR < 60 mL/min/1.73 m^2^		
	Odds ratio	95% CI	*P*
Age, rising	**1.08**	1.02–1.14	0.012
Hypertension	1.50	0.87–2.56	0.140
Diabetes mellitus	0.67	0.31–1.43	0.296
Sex: F versus M	1.97	0.87–4.49	0.105
Cardiovascular disease	**1.87**	1.15–3.03	0.011
Smoking	0.84	0.37–1.96	0.694
SBP	0.99	0.98–1.00	0.054
DBP	1.02	0.99–1.04	0.219
MBP	0.99	0.98–1.01	0.373
BMI, rising	0.97	0.93–1.03	0.357
WtHR, rising	**0.13**	0.00–0.66	0.031
LDL	0.95	0.40–2.26	0.915
Education	1.14	0.44–2.91	0.790

WOBASZ Senior: general population of the study: 918 (F 452, M 466); CKD population: 196 (F 129, M 67); age ≥75

*F* female, *M* male, *BMI* body mass index (kg/m^2^), *WtHR* waist-to-hip ratio, *SBP*, *DBP*, *MBP* systolic, diastolic, mean blood pressure; education other versus university

Bold letters—statistically significant

CKD prevalence in the studied population was higher in women, and female gender seemed to increase the chances of disease occurrence, though it was not a statistically significant result. In addition, a few risk factors which are usually analyzed in CKD population surveys, such as nicotinism or education level, proved to play no vital role.

## Discussion

Along with aging of European societies, common attention is paid to chronic diseases whose prevalence increases in the elderly, especially when their course is connected with deterioration of the quality of life, increase in mortality due to cardiovascular reasons and necessity of increasing financial expenses for medical care. It has been well demonstrated that there is a significant connection between CKD and the frequency of cardiovascular diseases and increased cardiovascular mortality in the general population, as well as in the elderly population [3, 17–21]. This fact seems to be the best, albeit indirect, answer to the voices questioning the validity of diagnosing CKD in elderly people. Being aware of the technical limits of eGFR estimation and the exceptions in the physiology of old age, the diagnosis of CKD that is made by a physician should be followed by the introduction of proper medical care and nephroprotective treatment. Epidemiological studies help in characterizing the group which will benefit the most from such a conduct. However, thus far, we have not had a lot of scientific data concerning CKD prevalence in elderly people in Poland. Our knowledge of this issue has been so far shaped by the results of the Polnef Study carried out in the Pomeranian region and published a few years ago, results of the nationwide Polsenior survey, as well as foreign surveys which are mostly American [6, 17, 22]. It is worth noting that a significant increase in CKD prevalence in elderly people was observed in the recently published NATPOL 2011 survey [5].

An essential advantage of this article is that we elaborate on and present results of a large epidemiological survey, which had a very advanced methodology of sampling, ensuring representativeness of the survey results for the Polish elderly population aged ≥75.

In our study, we have shown a very high CKD prevalence in the population in question (26.9%), which is consistent with earlier reports. Among the data presented by other authors, CKD prevalence varies from 21 to as much as 50%, depending on the methodology used and the precise age ranges. The authors of the Polsenior survey reported a slightly lower average value of decreased eGFR < 60 ml/min/1.73 m^2^ frequency (21.2%). However, the estimated incidence of CKD in the above-mentioned study was 29.4%, which may be as a result of it encompassing a younger group of respondents (≥65) and simultaneously taking into account assessment of albuminuria in the survey protocol [6].

We also assessed the awareness of participants regarding the prevalence of kidney disease. The obtained result of 17%, though generally low, is still higher than the one reported by the authors of the Polsenior survey, and even slightly higher than the one established in younger populations in the NATPOL 2011 survey. This may be due to Poles having relatively good accessibility to health services, and the educational and prophylactic role of basic health care. On the other hand, the elderly are characterized by relatively greater interest and concern for their health than younger people.

In the analysis of the percentage representation of the respective stages of CKD, special attention should be paid to the G3a category which is the predominant category and also raises the biggest controversies. Some authors doubt the clinical significance of diagnosing G3a stage CKD based on moderately lowered eGFR [23]. Moreover, as shown by Stengel et al. [24], there is a stronger connection between the G3b stage (in comparison with G3a) and cardiovascular diseases and deaths observed. As it has been established in earlier studies, we can observe a nonlinear decrease in eGFR values by, on average, 10 ml/min/1.73 m^2^ in a decade of life [4]. Therefore, it is possible that in the 75+ age group there are individuals in whom the decrease in eGFR to the level meeting CKD diagnosis results from the physiological aging of kidneys and is not a symptom of pathology. Thus, the debate concerning a possible lowering of the CKD diagnosis level in old age to the value of eGFR < 45 ml/min/1.73 m^2^, especially in situations where there are no other kidney damage markers, still remains open [25]. In our analysis, for example, it would mean a significant drop in the estimated number of CKD patients in the population from 495,590 to 148,356 people. At the moment, however, the opinion mentioned in the introduction of this article dominates—i.e., the definition and division of CKD is firmly supported by prevailing data, and the presented evidence is not sufficient to create a separate classification for the elderly population. Furthermore, elderly people can only benefit from improved care, prophylaxes and treatment that should follow the implementation of the current criteria [11].

In order to reaffirm the adequacy of the data presented in this study, the cited estimations were compared with the available data from the registry based on the actual number of patients in end-stage CKD. In the G5 category, there were up to 1.1% of all patients with CKD, which approximately gives a group of 5451 patients in end-stage renal disease. From the article “*The report on the state of renal replacement therapy in Poland”*, in 2007 exactly 958 patients in the age of ≥75 years started renal replacement therapy (RRT). Thus, the estimated number of patients in the G5 stage seems probable, as approximately 1/5 of the projected number entered the renal replacement therapy (RRT) program. It should be taken into account, that in the age range in question, there is nowadays a tendency to postpone the commencement of RRT. It should also be mentioned that, according to annual reports on RRT, in the subsequent years, an increasing percentage of those aged 75 and more were observed among patients on RRT. This could partly result from an increase in the number of kidney transplants in younger age groups and, thus, a percentage shift to the older population, but could also be the result of an aging society, in general, and the trend toward earlier qualification of these patients to RRT [26].

In our study population, a very high prevalence of arterial hypertension was noted. The result was predominantly dependent on the diagnosis established on the basis of declared hypotensive drug intake and, to a lesser extent, on the diagnosis established on the basis of blood pressure measurements taken. On the one hand, it proves wide accessibility of medical services to patients, and on the other hand, it raises fear of overdiagnosis—or rather—excessive AH therapy in old age. It should be remembered that therapeutic aims in AH in the population in question are much less rigoristic. Current Polish Society on Hypertension guidelines for patients above 80 years of age recommend a more careful lowering of systolic pressure to a level below 150 mmHg [27]. It should be noted that in the elderly the standard beneficial role of hypotensive drugs may become outweighed in the presence of CKD when inappropriately used (fixed combinations of diuretics may lead to dehydration, and ACEi/AT1 blockers may result in an increase in creatinine concentration).

In people with decreased eGFR, arterial hypertension was noted more often than in people with normal kidney function. However, unlike in people under 75 years of age, logistic regression analysis did not show any significant influence of AH on the increased risk of CKD occurrence. It was proven, however, that the occurrence of cardiovascular diseases in general definitely increased the risk of kidney disease (OR 1.87). It is worth mentioning that in the above-mentioned Polnef and Polsenior surveys, it was proven that there exists a significant influence of hypertension on the advancement of CKD. Perhaps, in this case it also arises from the analysis of a younger population—in both surveys a group of people ≥65 years of age was observed.

Surprising conclusions arose from the analysis of diabetes mellitus (DM) occurrence in the analyzed population. Both in people with normal, as well as in those with impaired renal function, DM prevalence was comparable and similar to that observed in the general elderly population. Logistic regression analysis showed that the presence of DM did not increase the risk of CKD in those aged 75+. This surprising occurrence is confirmed by the results of other authors. Shastri et al., based on results from a survey conducted among American octogenarians, also did not confirm the dependency of CKD and DM coexistence [17]. It should be added that, according to the authors of the NATPOL 2011 survey, DM and diabetic kidney disease are the most frequent causes of CKD in the general Polish population and the existence of DM triples the risk of CKD in people aged 18–79 years [5]. This evidently different epidemiology of this disease in the elderly may be drastically explained by the short life expectancy of diabetic patients who often do not survive to reach the old age. On the other hand, DM observed in the elderly is most likely a late-onset disease and, thus, has no substantial influence on the development of CKD. At this point, it is worth referring to the PRESAM survey, an epidemiological analysis published several years ago concerning the causes of end-stage renal failure [28]. The conclusions it reached were clear, that is, while diabetic nephropathy was the most common cause of this disease in the population between the ages of 45–65, in people above 70 years of age vascular nephropathy (defined by both—ischemic nephropathy and hypertensive kidney disease) took priority [29].

Besides the above-mentioned classic risk factors for CKD development, attention should also be turned to the close relationship between increasing age and the lowering of eGFR. Even more interesting seems to be the fact that, after attaining 75 years, increasing age no longer becomes a significant risk factor for CKD occurrence (OR 1.08). Therefore, we can expect that when a given person attains the age range in question with proper kidney function, the chances for significant deterioration of their function are minimal. This emphasizes the need to focus on appropriate health-promoting education and encouraging nephroprotective habits in younger people.

It is worth mentioning that we observed a reverse epidemiology for a classic CKD progression risk factor—the waist-to-hip ratio (WtHR). The WtHR is used to express and measure abdominal obesity [30]. In the analyzed 75+ population, it was found that an increase in the WtHR value may have a protective role, but only if it was within the normal reference range of this index. Such a link was not observed for the BMI. This phenomenon can be explained by the fact that the higher the WtHR, the lower the chances of cachexia or sarcopenia [31]. It needs to be stressed that this does not, however, imply that abdominal obesity provides any protection against CKD, as such a relationship has not been established for abnormally high WtHR values. On the contrary, in our analysis, the majority of people diagnosed with CKD were classified as obese on the basis of increased BMI values. Probably, what we observed is a similar phenomenon to that of patients treated with repeated dialysis, in whom reverse epidemiology of these risk factors was observed, in terms of their impact on morbidity and mortality [32, 33]. It should be pointed out that the existence of a reversed relation in this scope (i.e., WtHR) was also observed in elderly people elsewhere [34, 35].

In view of the anticipated methodology provided for in the WOBASZ survey of determining creatinine concentration through Jaffe’s kinetic method, a decision was taken to estimate values of eGFR based on the simplified MDRD formula (the now commonly used CKD-EPI formula is based on the measurement of serum creatinine using the enzymatic IDMS traceable technique) [36]. As a result of the use of this equation, credible results were obtained for the values of eGFR < 60 mL/min/1.73 m^2^, and this resulted in us being able to take into account CKD from the G3a stage upwards in our analysis. An additional reason for such a choice was the fact that in the outline of this survey other markers of kidney damage, e.g., albuminuria, were not included. Undoubtedly, this is an undeniable drawback of the presented analysis. Nonetheless, by comparing Polnef (based primarily on the determination of the level of albuminuria) and Polsenior (which used both albuminuria and eGFR) surveys, we can go as far as to assume that in the analyzed age group the main component of CKD diagnosis is the decrease in eGFR, while the occurrence of albuminuria (in contrast to younger age groups) plays a minor role.

We realize that the presented results may have flaws typical for population-based cross-sectional studies in this field, which stem from the application of the simplified definition of CKD, only a single determination of creatinine level, and the absence of image studies. However, in favor of the presented analysis is a credible methodology of sampling of the representative population, a relatively high percentage of obtained results, as well as convergence with results published by other researchers. Therefore, it should be assumed that the presented results show the actual situation of CKD prevalence in those aged 75 years and above in the Polish population.

## Conclusion

In the presented work, it was shown that CKD prevalence is high in the Polish population aged 75+ and is at 26.9% (~495,590 patients), whereas awareness of the disease is relatively low. Both AH as well as increasing age did not essentially increase the risk of CKD in the examined population. It was observed, however, that there is a substantial difference, in the prevalence of DM among CKD patients, compared to that of a younger population. In CKD patients, the prevalence of DM was close to that of the general elderly population.
